# Exploring emotional expression recognition in aging adults using the Moving Window Technique

**DOI:** 10.1371/journal.pone.0205341

**Published:** 2018-10-18

**Authors:** Elina Birmingham, Joakim Svärd, Christopher Kanan, Håkan Fischer

**Affiliations:** 1 Faculty of Education, Simon Fraser University, Burnaby, BC, Canada; 2 Department of Psychology, Stockholm University, Stockholm, Sweden; 3 Chester F. Carlson Center for Imaging Science, Rochester Institute of Technology, Rochester, NY, United States of America; University of Hong Kong, HONG KONG

## Abstract

Adult aging is associated with difficulties in recognizing negative facial expressions such as fear and anger. However, happiness and disgust recognition is generally found to be less affected. Eye-tracking studies indicate that the diagnostic features of fearful and angry faces are situated in the upper regions of the face (the eyes), and for happy and disgusted faces in the lower regions (nose and mouth). These studies also indicate age-differences in visual scanning behavior, suggesting a role for attention in emotion recognition deficits in older adults. However, because facial features can be processed extrafoveally, and expression recognition occurs rapidly, eye-tracking has been questioned as a measure of attention during emotion recognition. In this study, the Moving Window Technique (MWT) was used as an alternative to the conventional eye-tracking technology. By restricting the visual field to a moveable window, this technique provides a more direct measure of attention. We found a strong bias to explore the mouth across both age groups. Relative to young adults, older adults focused less on the left eye, and marginally more on the mouth and nose. Despite these different exploration patterns, older adults were most impaired in recognition accuracy for disgusted expressions. Correlation analysis revealed that among older adults, more mouth exploration was associated with faster recognition of both disgusted and happy expressions. As a whole, these findings suggest that in aging there are both attentional differences and perceptual deficits contributing to less accurate emotion recognition.

## Introduction

### Facial expression recognition and adult aging

The ability to read emotion information from faces declines as a function of increasing adult age. However, these deficits are more pronounced for certain facial expressions (e.g., [1]). As shown in a meta-analysis by Ruffman, Henry, Livingstone and Phillips [2], recognition of anger, fear and sadness was most affected by age, while recognition of surprise and happiness was less so. In contrast, there was also a trend for older adults (OA) to be better than younger adults (YA) in recognizing disgust [2]. This suggests that OA have difficulties recognizing some facial expressions, but not all. It also indicates that OAs may have more difficulties in recognizing expressions with diagnostic features situated in the top-half of the face (e.g., the eyes in fear and anger) than for expressions with diagnostic features situated in the bottom-half of the face (e.g., nose and/or mouth in happiness and disgust [3].

### Attention allocation and the facial expression recognition

Based on eye-tracking research with younger adults, there is evidence that facial expression recognition ability is dependent on the capacity to allocate visual attention efficiently. For instance, Shurgin end colleagues [4] found that the eyes were fixated proportionally more on facial expressions of anger, fear, sadness, and shame than on disgusted and happy faces. In contrast, participants fixated the mouth proportionally more on disgusted and happy faces relative to angry and sad faces [4]. In a now classic study, Adolphs et al. (2005) reported that amgydala damaged patient SM’s failure to recognize fear was driven by a lack of fixations to the eye region, and her fear recognition dramatically improved when she was instructed to fixate the eyes [5]. Similarly, Corden and colleagues found that poor recognition of fear in adults with Asperger syndrome was correlated with low fixations to the eye region [6]. These findings are supported by research showing that the eyes and adjacent areas (e.g., eyebrows) are the most diagnostic features in recognition of fear [7–9], and anger [7–8], while the mouth is more informative in the recognition of happiness [7–8], surprise [8], and disgust [7–8]; but see also [9].

Thus, it can be concluded that recognition of fear and anger is facilitated by attending to information from the top part of the face, while recognition of happiness and disgust is more dependent on attending to information from the bottom part of the face.

### Age differences in scanning of facial expressions

Visuo-attentional patterns have also been acknowledged in the aging and emotion literature as a potential explanation for age differences in recognition accuracy. Several eye tracking studies have shown that compared to YA, OA spend less time looking at the top half of the face relative to YA when asked to judge facial expression (e.g., [10, 11, 12, 13], but see also [14] or are less likely than YA to initially fixate the eye region [15]. More specifically, these age differences in scanning patterns have been shown when viewing angry [11, 13], disgust [10, 12], fearful [10, 11, 12, 13], happy [10], sad [10–11, 13], surprised [12], and neutral [10, 13, 16] faces. Thus, a global reduction in attention allocation to the top of the face could account for reduced recognition of expressions for which recognition relies primarily on information from the eyes. Instead, OA seem to rely on the bottom part of the face, sparing recognition of emotions for which recognition relies primarily on the mouth.

These findings allow predictions about adult age-related emotion recognition and differences in visual scanning patterns. First, because OA attend relatively more to the bottom of the face and less to the top of the face, relative to YA, OA should show reductions in accuracy for expressions like anger and fear, and better (or spared) performance on expressions like disgust (and possibly happiness). This is the general pattern reported in the meta-analysis by Ruffman et al. [2]. Critically, if visuo-attentional differences (as opposed to more fundamental difficulties in visual perception) are responsible for this pattern, one would expect a similar pattern at an individual level. That is, those older adults who spend more time fixating the most diagnostic region for a specific emotion will perform better than those who fail to attend to those regions. Consistent with this predicted pattern, Wong et al. [13] showed that (a) OA were less accurate than YA at recognizing angry, fearful, and sad faces, but more accurate than YA at recognizing disgusted faces; and (b) for angry, sad, and fearful expressions (“top expressions”), both OA and YA benefited from spending relatively more time exploring the top part of the face. Finally OA, but not YA, benefitted from exploring the bottom part of the face when viewing disgusted faces. In contrast, Sullivan et al. [12] reported that for OA, looking at the mouth correlated with *worse* performance, even for disgust and happiness (“bottom expressions”). In addition, these authors found that exploration time on the eyes was associated with better accuracy for fearful and angry expressions (“top expressions”) in YA, but not OA. Finally, while Circelli et al. [10] found the predicted scanning pattern (OA spent relatively more time on bottom vs. top regions of face compared to YA), they found no correlations between the scanning patterns of either OA or YA and recognition performance.

It is important to note that these studies are all based on eye-tracking methodology, a technique with some distinct limitations for measuring the allocation of attention.

### Limitations of eye tracking methodology

While eye tracking is widely used to index the locus of overt attention, the assumption that foveal vision sets a boundary for that locus is questionable. Indeed, it has been demonstrated that, although overt orienting (i.e., orienting accompanied by eye movements) and covert orienting (i.e., orienting without eye movements) are linked, they are separable [17]. This decoupling of overt and covert attention is reflected by findings showing that diagnostic features (i.e., the eyes and the mouth) are available extrafoveally. For example, Caldara and colleagues found that although East Asian participants fixated more on the nose and less on the eyes compared to Western Caucasian participants in an unrestricted vision condition, their ability to recognize faces was unaffected [18]. Importantly, in two gaze-contingent conditions with a restricted viewing window (2° and 5°), both groups fixated on the eyes and the mouth to the same extent, suggesting that both groups attended to the same facial information, despite differences in their fixation patterns in the unrestricted visual field condition. In a separate line of research, Calvo and Esteves [19] found high detection accuracy for both foveally and parafoveally presented emotional expressions. Combined, these studies show that important diagnostic information can be gathered outside foveal attention. Taken together, because allocation of visual attention is not restricted to the point of fixation, one must question the utility of eye tracking as an index of visual information use during emotion recognition.

Second, faces are processed rapidly and with a small number of fixations. Both computational [20] and human experimental studies [21] have shown that two fixations are sufficient for recognizing previously seen faces. Similarly, stimulus exposure times as low as 25 msec [19, 22] and detection times as low 160 msec [23] have been associated with reliable emotional recognition. Given the speed with which faces are processed, it is noteworthy that most eye-tracking studies of emotion recognition report much longer stimulus durations, ranging from 2–4 seconds [5, 12–13]. The discrepancy between the presentation time and the time needed for emotion recognition suggests that task-irrelevant eye movements are being captured in many of the studies reported in the previous section.

### The Moving Window Technique

**Fig 1:**
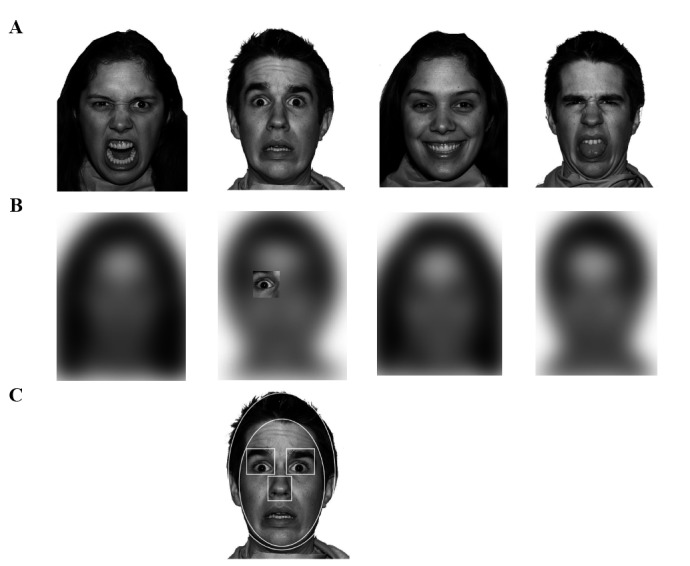
**(A)** Example of unblurred faces showing anger, fear, happiness, and disgust. **(B)** Example of the same expressions blurred and the movable window. **(C)** The regions of interest. Note, publication of the NimStim models included in this Fig is in accordance with the terms of agreement for the stimulus set, which can be found at http://www.macbrain.org/resources.htm.

In our study, we used the Moving Window Technique (MWT) [24] to study emotional expression recognition. MWT is an alternative to eye tracking, and it avoids many of the shortcomings described earlier. In MWT, the participant is presented with a blurred image, which in our study is a face. Using the mouse, the participant controls a small window that removes the blur from the image region within the window (see Fig 1 for an example). Two features of MWT allow for a more direct measure of visual information attended to during emotion recognition. First, by restricting the visual field to a moveable window, the MWT prevents the rapid and early extraction of visual information relevant to emotion recognition. Indeed, since the entire face is never available to the participant, he/she is forced to explore the face by moving the window around. Second, exploration is terminated as soon as the participant gives a response, reducing task-irrelevant exploration data. The MWT has been used to reveal important age-related differences in face exploration from young childhood to young adulthood [24], and here we apply the same technique to examine age-related changes in older adults.

### Aim

Using the MWT, our aim was to identify differences in exploration patterns between OA and YA that may contribute to performance on a facial emotion recognition task. We hypothesized that: (1) compared to YA, OA would spend less time on the eyes (upper regions of the face) relative to YA, but more time than YA on the mouth and nose (lower regions of the face), (2) this exploration strategy would be reflected in greater age-related deficits in recognition of fear and anger (“top expressions”) than in recognition of happiness and disgust (“bottom expressions”), (3) at the individual level, more eye exploration is expected to be associated with better recognition of top-expressions; and more mouth viewing would be associated with better recognition of bottom-expressions.

We were secondarily interested in the extent to which age-related differences in eye exploration could be localized to either the left eye (i.e., the eye in the left visual field of the participant) or the right eye (i.e., the eye in the right visual field of the participant), because in previous research [24] we found that young adults show more left-eye exploration relative to children during emotion recognition. This preference for the left-eye in adulthood led to a left-eye bias (more exploration of the left eye than the right eye) that may reflect maturation of right-lateralized face processing areas in the brain [25–28]. We wondered if this left-eye attentional bias might be altered in aging adults, a question that to our knowledge is unexplored.

## Methods

### Participants

Forty-two (22 females; 20 males) young adults and 42 (20 females; 22 males) older adults were recruited through advertisements in local newspapers. The participants reported normal or corrected-to-normal vision, no current or previous psychological treatment, or drug abuse. Groups were matched on cognitive ability (via the Mini Mental State Examination test [29]), and years of education (*ps* > .05), see Table 1. Participants were compensated with a lottery ticket worth $8 USD for study completion. The Stockholm University ethics committee approved the study, and written informed consent was received from each participant.

**Table 1:** Demographic information for each participant group. Values are means ± standard deviations.

	Younger Adults	Older Adults
**Age (years)**	23.9 ± 2.8	69.1 ± 3.9
**MMSE**	29.3 ±.8	29.4 ± .7
**Years of education**	14.9 ± 2.3	14.7 ± 3.1

MMSE = Mini Mental State Examination (Folstein et al., 1975 [25])

### Materials

Ten actors (5 female, 5 male) from the NimStim set of facial expressions [30] depicted each of four expressions (anger, fear, happiness, disgust), resulting in a total of 40 trials (see Fig 1 for examples of the stimuli). The forty images were blurred with a Gaussian filter (with a standard deviation equal to 40 pixels, corresponding to approximately 12% of the face width) so that expression recognition was not possible without moving the window around (described in Procedure). Face images were displayed on a white background measuring 1280 x 800 pixels in size, subtending 27 x 17 degrees of visual angle (°) at a viewing distance of approximately 60 cm. The images were presented with Matlab R2012 on a 13” MacBook Pro.

### Procedure

Before the experiment began, an introductory screen containing a picture of each expression (not included in the experiment) was presented on the screen with corresponding emotion labels beneath the faces. This screen introduced the participants to the four possible expressions, and familiarized them with the response buttons. Next, participants were informed that they were about to see a blurry face, which they could explore through a moveable window in which the face was not blurred. This 100 x 100-pixel window (subtending 2.14° x 2.14° and approximately 4% of the face) was initially positioned in the center of the face in the beginning of each trial (See Fig 1C). After this, the window position was fully controlled by the position of the mouse. Participants were told that they would have up to 15 seconds to explore the face, but that they should respond as soon as they recognized the emotion by pressing the space bar, at which time the trial was terminated and replaced by a response screen. Participants then used one of four labeled keys to enter their response (Angry, Happy, Disgusted, Scared). After the response was entered, the trial was terminated and the next trial was initiated. For each trial, accuracy (correct/incorrect), response time (RT), and mouse coordinate data sampled at a rate of 60 Hz were recorded. Four practice trials were carried out prior to the actual experimental trials. Presentation order of the 40 trials was randomized across subjects.

### Data preparation and analysis

Three participants (2 older women, 1 younger man) were excluded from the analysis due to abnormal response times (> 2 SD from the mean). The faces were divided into six mutually exclusive regions of interest (ROI); left (from the perspective of the participant) eye, right eye, nose, mouth, face remainder (minus internal features) and hairline/ears (See Fig 1C for an example). From the data, we analyzed five variables:

1. *Accuracy*–the percentage of correct responses.
2. *Response time*—latency between onset of the face image and key press to end the trial) for correctly recognized faces. This value also reflects the time taken to explore the face before responding.
3. *Percentage of exploration time* spent on each of the 6 ROIs—a value that reflects both the time and the amount of ROI information (pixels) revealed by the window during exploration. The percentage of exploration time within each ROI was normalized by ROI size to correct for the fact that bigger ROIs would, by definition, have greater overlap with the window than smaller ROIs [24]. We also prepared exploration heat maps visualizing the exploration data over time (1^st^ third, 2^nd^ third, and 3^rd^ third of the viewing session) for each age group separately.
4. *The last ROI explored prior to response*, i.e., the ROI in which the center of the window was positioned before the spacebar was pressed to end the trial; for each participant we computed the % of trials on which the last ROI was on the left eye, right eye, nose, or, mouth. We reasoned that this measure might reveal interesting age-related differences in ROI exploration at the time of emotion judgment and subsequent response.
5. *Correlations between an eyes-mouth difference score and recognition performance* (accuracy and RT) within each age group; eye-mouth difference scores were created at an individual level by subtracting the (area-corrected) percentage of time spent on the mouth from the percentage of time spent on the eyes (combined).

Prior to analysis, the RT-data were log-transformed to achieve normal distribution. However, to facilitate interpretation of the results, untransformed values are reported in tables and figures. In all analyses, uncorrected degrees of freedom are reported together with observed significance levels after Greenhouse-Geisser correction. Results were considered significant at *p* ≤ .05.

## Results

### Accuracy of emotion recognition

Means and standard deviations for accuracy and RT are presented in Table 2. An ANOVA on *accuracy*, with age (YA, OA) as the between-subjects factor and emotion (anger, fear, happy, disgust) as the within-subjects factor revealed a main effect of emotion, *F*(3, 237) = 17.40, *p* < .001, η^2^ = .180. Pairwise comparisons (Sidak adjustment for multiple comparisons) showed that recognition was highest for happy (*M* = 97.6%) and fearful faces (*M* = 97.0%), which did not differ (*p* = .98), and lower for angry (*M* = 90.7%) and disgusted faces (*M* = 90.4%), which did not differ (*p* = 1.00). Happy and fearful faces were each recognized more accurately than angry faces (happy vs. angry, *p* < .001; fearful vs. angry, *p* < .001); happy and fearful faces were also recognized more accurately than disgusted faces (happy vs. disgusted, p < .001; fearful vs. disgusted, p < .001).

**Table 2:** Mean (SD) accuracy and response time performance by facial expression and age group.

	Anger	Fear	Happy	Disgust
**Accuracy (% correct)**				
Younger adults	92.20 (10.37)	98.54 (4.22)	99.02 (3.00)	95.61 (9.50)
Older adults	89.00 (11.72)	95.50 (8.46)	96.25 (5.89)	85.00 (13.96)
	*d* = -0.29	*d* = -0.46*	*d* = -0.60**	*d* = -0.89***
**Response time (sec)**				
Younger adults	3.01 (0.17)	2.74 (0.16)	2.05 (0.09)	2.83 (0.16)
Older adults	5.32 (0.28)	5.54 (0.26)	3.86 (0.26)	5.91 (0.33)
	*d* = 1.56***	*d* = 2.01***	*d* = 1.49***	*d* = 1.87***

Effect size (Cohen’s d) and significance (*p < .05, **p < .01, ***p < .001) of the group difference is also presented.

A main effect of age, *F*(1, 79) = 17.89, *p* < .001, η^2^ = .185, demonstrated that across expressions, older adults were less accurate than younger adults. These effects were qualified by an emotion by age interaction, *F*(3, 237) = 3.98, *p* = .014, η^2^ = .048. Planned 2-tailed t-tests (p-values adjusted for unequal variances) indicated that OA were less successful than YA in recognizing fearful, *t*(79) = 2.04, *p* = .046, happy, *t*(79) = 2.67, *p* = .010, and disgusted, *t*(79) = 3.99, *p* < .001, faces, but no group difference was found for angry faces, *t*(79) = 1.30, *p* = .197 . As we will discuss, this pattern does not fit with our prediction (#2) that OA would show the largest deficits in emotion recognition for “top expressions” (i.e., anger and fear) and spared performance for “bottom expressions” (i.e., disgusted and happy faces). Indeed, Table 2 shows that the largest group difference in accuracy was for disgusted faces.

### Response time

The ANOVA on response times (RT), with age (YA, OA) as the between-subjects factor and emotion (anger, fear, happy, disgust) as the within-subjects factor revealed a main effect of emotion, *F*(3, 237) = 85.69, *p* < .001, η^2^ = .520, reflecting that happy faces were recognized faster than the other expressions (pairwise comparisons *ps* < .001), which did not differ in RT (*ps* > .35). A main effect of age, *F*(1, 79) = 92.70, *p* < .001, η^2^ = .540, indicated that OA were slower to respond than YA (*p* < .001). An emotion by age interaction, *F*(3, 237) = 4.85, *p* = .003, η^2^ = .058 and pairwise comparisons reflected that, although the groups differed for all emotions (*t*_*anger*_(79) = 7.67, *p* < .001; *t*_*fear*_(79) = 9.49, *p* < .001; *t*_*happy*_(79) = 8.55, *p* < .001; *t*_*disgust*_(79) = 9.10, *p* < .001), the group differences were slightly larger for fearful and disgusted faces. Thus, while older adults needed more time to explore all faces before correctly identifying their emotion, we did not find systematic evidence that group differences in performance were minimized for “bottom expressions” (i.e., happy and disgust).

### Percentage of exploration time in each ROI

**Fig 2:**
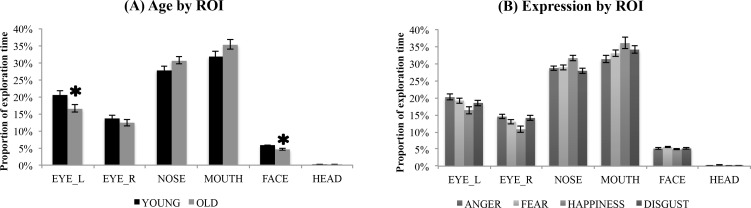
**(A)** percentage of exploration time by regions of interest and age group; * indicates a signficant group difference (*p* < .05). **(B)** percentage of exploration time by regions of interest and emotion. YA = younger adults; OA = older adults; Error bars represent standard error of the mean. Note FACE = face remainder (face minus eye_L, eye_R, nose, mouth); HEAD = hairline/ears.

Means and standard deviations for area-normalized percentage of exploration time in each ROI are visualized in Fig 2. The data were submitted to an ANOVA on percentage of exploration time, with age (YA, OA) as the between-subjects factor, and emotion (anger, fear, happy, disgust) and ROI (left eye, right eye, nose, mouth, face remainder, hairline/ears) as within-subjects factors. The ANOVA revealed a main effect of ROI, *F*(5, 395) = 312.23, *p* < .001, η^2^ = .798, reflecting that overall, the mouth was explored for the largest proportion of time (*M* = 33.6%), followed by the nose (*M* = 29.3%), with the two ROIs differing (*p* < .05). Mouth and nose were each explored for proportionally longer than all other regions (*ps* < .001). In addition, the left eye (*M* = 18.6%) was explored for proportionally longer than the right eye (*M* = 13.1%), *p* < .001; and both eye ROIs were explored for longer than the face remainder (*M* = 5.20%) and hairline/ears (*M* = 0.20%), *p*s < .001.

This main effect was qualified by an age x ROI interaction, *F*(5, 395) = 3.52, *p* = .004, η^2^ = .043 and an emotion by ROI interaction, *F*(15, 1185) = 12.76, *p* < .001, η^2^ = .139, but no three-way interaction (*F*(15,1185) = 1.11, *p* = .346, η^2^ = .014). Separate ANOVAs on each ROI were conducted to follow up the two-way interactions. The follow-up analysis on the ROI by age interaction revealed that across expressions, OA spent less time than YA viewing the left eye, *F*(1, 79) = 5.78, *p* = .019, η^2^ = .068, but not the right eye, *F*(1, 79) < 1, *p* = .355, η^2^ = .011. OA also spent less time than YA exploring the face remainder, *F*(1, 79) = 9.26, *p* = .003, η^2^ = .105, but not the hairline/ears, *F*(1, 79) < 1, *p* = .521 η^2^ = .005. Marginally consistent with our predictions, OA showed trends toward more time than YA exploring the mouth, *F*(1, 79) = 2.92, *p* = .091, η^2^ = .036, and nose, *F*(1, 79) = 3.94, *p* = .051, η^2^ = .048. No age by emotion interaction reached significance for any of the ROIs (*p*s all > 0.20), suggesting that age-related differences in exploration strategy were consistent across expressions.

Following up on the age-related reduction in exploration of the left eye, we explored whether the left-eye bias (more exploration of the left eye vs. the right eye) was present in each age group. Paired t-tests (2-tailed) revealed that both YA and OA explored the left eye proportionally more than the right eye, for each emotion (**YA**: anger, *t*(40) = 4.06, *p* < .001; fear, *t*(40) = 4.04, *p* < .001; happy, *t*(40) = 2.85, *p* < .01; disgust, *t*(40) = 2.79, *p* < .01; **OA**: anger, *t*(39) = 2.59, *p* < .05; fear, *t*(39) = 4.69, *p* < .001; happy, *t*(39) = 3.08, *p* < .01; disgust, *t*(39) = 2.81, *p* < .01). Thus, even though OA showed a reduction in left-eye exploration relative to YA, the left-eye bias was still present for OA. Independent t-tests comparing the magnitude of the left eye bias between YA and OA failed to reveal group significant differences (angry, *t*(79) = 1.78, *p* = .08; fear, *t*(79) = 1.33, *p* = .19; happy, *t(*79) = .48, *p* = .63, disgust, *t*(79) = .95, *p* = .35).

The follow-up to the ROI X emotion interaction revealed main effects of emotion on each ROI (left eye: *F*(3, 237) = 11.38, *p* < .001, η^2^ = .126; right eye: *F*(3, 237) = 15.59, *p* < .001, η^2^ = .165; mouth: *F*(3, 237) = 9.84, *p* < .001, η^2^ = .111; nose: *F*(3, 237) = 20.11, *p* < .001, η^2^ = .203; face remainder: *F*(3, 237) = 11.19, *p* < .001, η^2^ = .187) except for hairline/ears (*F*(3, 237) = 1.93, *p* = .125, η^2^ = .024). Pairwise comparisons revealed that the left eye was explored for longer on angry (*M* = 20.2%) and fearful (*M* = 19.2%) faces relative to happy (*M* = 16.4%) faces, *p*s < .05; angry and fearful faces did not differ, *p* = .23. The left eye was also explored more on angry faces than on disgusted faces (*M* = 18.5%, p < .01), but the difference between fearful and disgusted faces was not significant (*p* = .700), nor was the difference between happy vs. disgusted faces (*p* = .077). Similarly, the right eye was explored for longer on angry (*M* = 14.5%) and fearful (*M* = 13.0%) faces than on happy faces (*M* = 10.8%, *p*s < .01) but not relative to disgusted faces (*M* = 14.1%; *p*s>.20). Angry and fearful faces significantly differed on right eye exploration (angry > fearful; *p* < .05). Finally, the right eye was explored longer on disgusted than on happy faces (*p* < .001). For the mouth, happy faces received the most exploration on this ROI (*M* = 36.0%), with significant differences relative to angry (*M* = 31.3%, *p* < .001), fearful (*M* = 33.0%, *p* < .05), but not disgusted faces (*M* = 34.1%, *p* = .31). Disgusted mouths were explored longer than angry mouths (*p* < .001) but not fearful mouths (*p* = .62). The nose ROI was explored for longer on happy faces (*M* = 31.6%) than on all other expressions (*p*s < .001), which did not differ (*p*s > .10; angry: *M* = 28.7%, fearful: *M* = 28.9%; disgusted: *M* = 27.9%). Finally, the face remainder, while explored relatively sparingly, was explored longer on fearful faces (*M* = 5.6%) than on all other faces (angry: *M* = 5.1%; happy *M* = 4.9%, disgusted: *M* = 5.2%), *p*s < .001. No other differences were significant (*p*s > .05).

**Fig 3:**
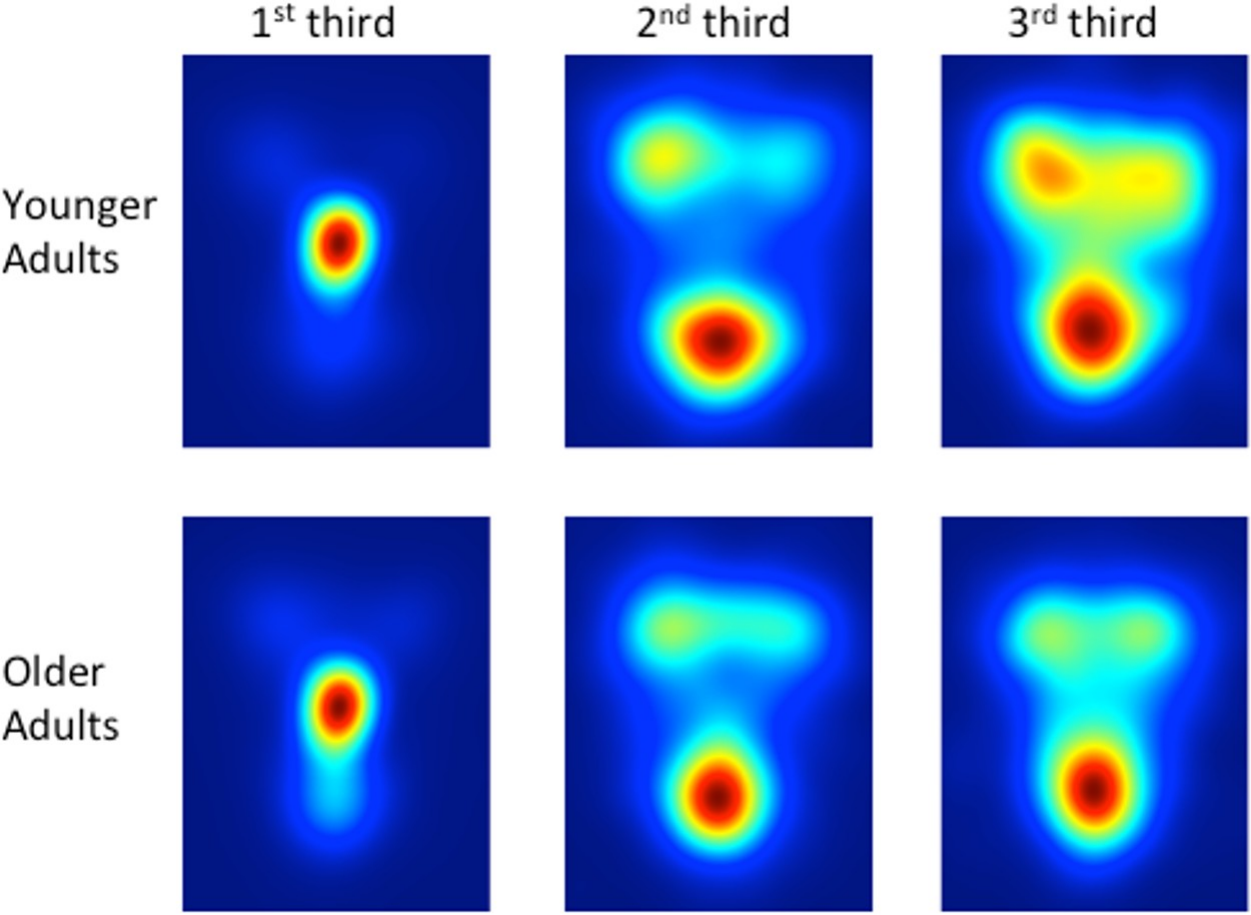
Exploration heat maps showing location of exploration time as a function of age group and time course (1^st^ third, 2^nd^ third, 3^rd^ third) of the exploration session. Data are pooled across all emotions, since the ROI analysis did not reveal any interactions between age and emotion. Mouse exploration data (x,y) were weighted by exploration time and smoothed with a 48-pixel Gaussian filter. Each individual heat map is scaled from zero time (blue) to the point of maximal exploration time (red).

Fig 3 depicts heat maps that visualize exploration patterns in each age group over time. We divided the exploration data into 3rds, for each participant separately, and then created pooled heat maps across participants for the 1^st^ third, 2^nd^ third, and 3^rd^ third of exploration. Data were pooled across all emotions, since the ROI analysis did not reveal any interactions between age and emotion with respect to exploration patterns. Mouse exploration data (x,y) were weighted by exploration time and smoothed with a 48-pixel Gaussian filter. Each individual heat map is scaled from zero time (blue) to the point of maximal exploration time (red).These maps illustrate the general pattern found by the ROI analysis, with the added information that both groups start out exploration on the nose (where the window is positioned at the start of the trial), and that over time, particularly by the 3^rd^ third of exploration, YA show relatively more exploration on the eyes relative to OA.

Overall, these data partially supported our prediction (#1) that OA would spend less time than YA exploring the upper regions of the face (the eyes)–with the nuance that this reduction in exploration time was specific to the left eye. However, we found only marginal evidence for increased exploration of the lower regions of the face (nose, mouth) in OA relative to YA.

### Last ROI to be explored

**Fig 4:**
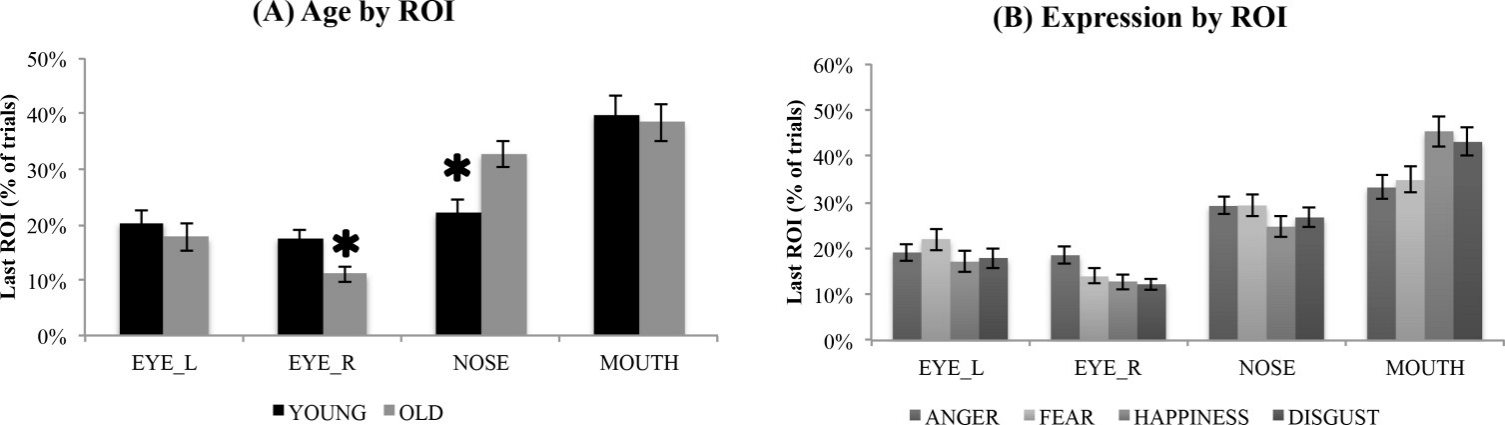
Last ROI to be explored. **(A)** percentage of trials on which exploration ended (i.e., position of the moving window at the end of the trial) on the left eye, right eye, nose, or mouth, broken down by age group; * indicates a signficant group difference (*p* < .05). **(B)** percentage of trials on which exploration ended on the left eye, right eye, nose, or mouth, broken down by emotion. YA = younger adults; OA = older adults; Error bars represent standard error of the mean.

We reasoned that examining the very last ROI to be explored might reveal important group differences in facial information use at the time of response execution (spacebar pressed to end the trial and enter emotion judgment). For this analysis we focused on the internal features of the face (left eye, right eye, nose, mouth) that are diagnostic for facial expression recognition (see Fig 4). The ANOVA revealed a main effect of ROI, *F*(3,237) = 27.91, *p* < .001, η^2^ = .261, reflecting that the mouth (*M* = 39.1%) was most frequently the last ROI to be explored (more than all other ROIs, *p*s all < .05). The nose (*M* = 27.5%) was more likely to be the last ROI explored than either eye (left eye: *M* = 19.0%, right eye: *M* = 14.4%, *p*s < .01). Finally, the two eyes did not differ (*p* = .09).

The main effect of ROI was qualified by a significant age x ROI interaction, *F*(3,237) = 3.26, *p* = .022, η^2^ = .040, and an emotion x ROI interaction, *F*(9,711) = 4.87, *p* < .001, η^2^ = .058. The 3-way interaction was not significant, *F*<1. Follow up ANOVAs on each ROI were conducted to explore the interaction effects. For the left eye, there were no main effects of emotion or age, nor any interaction (*p*s > .10). For the right eye, however, there was a main effect of emotion, *F*(3,237) = 3.83, *p* < .01, η^2^ = .046, reflecting that participants were more likely to end their exploration on the right eye for angry expressions than for all other expressions (*p*s < .05), with no other significant differences (*p*s >.30). In addition, a main effect of age, *F*(1,79) = 9.16, *p* < .01, η^2^ = .104, reflected that OA were less likely than YA to end their exploration on the right eye (OA *M* = 11.1%; YA *M* = 17.6%, *p* = .003). The age x emotion interaction was not significant, *F*<1. For the mouth ROI, a main effect of emotion, *F*(3, 237) = 9.63, *p* < .001, η^2^ = .109, reflected that the last ROI was more likely to be the mouth on fearful (*M* = 43.1%) and disgusted (*M* = 45.4%) faces than on angry (*M* = 33.2%) or happy (*M* = 34.8%) faces, *p*s < .01. The effect of age was not significant (*F*<1), nor was the interaction, *F*(3,237) = 1.17, *p* = .32, η^2^ = .015. Finally, for the nose ROI, a main effect of age, *F*(1,79) = 9.94, *p* < .01, η^2^ = .112, indicated that OA were more likely than YA to end their exploration on the nose (OA *M* = 32.8%; YA *M* = 22.1%, *p* = .002). The effects of emotion and the age x emotion interaction were not significant (*ps* > .10).

Thus, again this pattern reveals some differences in exploration strategy associated with aging, whereby OA were less likely than YA to end their exploration on the right eye, and more likely than YA to end their exploration on the nose. However, we did not find an age-related bias to end exploration on the mouth; indeed, both groups were highly likely to end exploration on the mouth, suggesting that this ROI may have been particularly informative for emotion recognition.

### Correlations between eyes-mouth difference score and accuracy/RT

**Fig 5:**
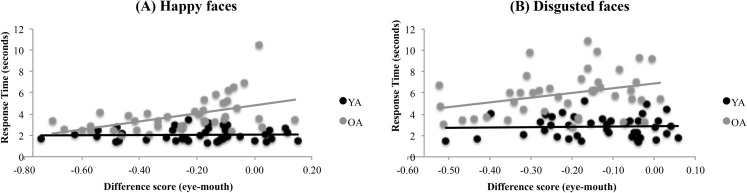
Scatter plots of eyes-mouth % difference scores vs. RT for **(A)** happy faces, **(B)** disgusted faces. Positive values on the x-axis represent more eye-viewing; negative values represent more mouth-viewing. Lower values on the y-axis indicate shorter response time (i.e., faster detection of emotion).

To compute the eyes-mouth difference score, we subtracted the % of exploration time on the (combined) eyes from the % of exploration time on the mouth, separately for each subject. A positive difference score thus indicates a preference for eyes over mouths; a negative score indicates a preference for mouths vs. eyes. For OA (but not YA), relative mouth preference was associated with faster correct identification of happy, *r*(40) = .53, *p* < .001), and disgusted, *r*(40) = .33, *p* < .05), expressions (see Fig 5). However, no other correlations were significant (see Table 3). Thus, we found partial evidence for our prediction (#3) that at the individual level, more eye exploration should be associated with better (i.e., faster) recognition of top-expressions; and more mouth viewing should be associated with better recognition of bottom-expressions.

**Table 3:** Pearson correlations (*r*) between eyes-mouth % difference scsore, correct RT, and accuracy for each emotion condition.

	Anger	Fear	Happy	Disgust
		**Accuracy**		
eyes-mouth%	YA = .089	YA = -.126	YA = -.178	YA = .044
	OA = .003	OA = .067	OA = .068	OA = -.118
		**Response Time**		
eyes-mouth%	YA = -.212	YA = .041	YA = .024	YA = .054
	OA = .251	OA = -.037	OA = .534**	OA = .326*

YA = younger adults; OA = older adults.

* *p* < .05,

** *p* < .001 (2-tailed)

## Discussion

The aim of this study was to identify differences in exploration patterns between older adults and younger adults that may contribute to facial emotion recognition deficits associated with aging. While some previous research (e.g., [13]) has suggested that differences in attention allocation contribute to impaired emotion recognition performance in older adults (OA), there are inconsistencies in the literature (e.g., [10, 12]). Previous studies used eye tracking as a proxy for attention, which, while informative, may have led to inconsistent findings because a considerable amount of facial information is extracted rapidly outside the fovea. Thus, we used the MWT, a method that restricts the visual information to a small moveable window, as a measure of visual attention to facial features during emotion recognition.

We hypothesized that: (1) compared to younger adults (YA), OA would spend less time on the eyes (upper regions of the face) relative to YA, but more time than YA on the mouth and nose (lower regions of the face); (2) this exploration strategy would be reflected in greater age-related deficits in recognition of fear and anger (“top expressions”) than in recognition of happiness and disgust (“bottom expressions”); and (3) at the individual level, more eye exploration was expected to be associated with better recognition of top-expressions, and more mouth viewing should be associated with better recognition of bottom-expressions.

We found partial support for these hypotheses. First, while YA spent proportionally more time than OA exploring the eyes for information during facial emotion recognition, OA spent only marginally more time than YA exploring the mouth and nose. The present study failed to support our second prediction, that OA’s overall enhanced exploration of the mouth would be reflected in a greater group-level deficit in the recognition of fear and anger (top expressions) than for happiness and disgust (bottom expressions). Instead, we found that OA were less successful than YA in recognizing fearful (top), happy (bottom), and disgusted (bottom) expressions, but performed similarly to YA in recognizing angry (top) expressions. Indeed, OA showed the strongest detriment in accuracy for disgusted faces, in stark contrast to previous work showing that OA are spared, or even slightly superior relative to YA, at recognizing disgust [2]. Reduced accuracy for disgusted faces was especially surprising given an overall high level of mouth exploration in both groups and the (nonsignificant) trend towards more mouth and nose exploration in OA relative to YA. However, at the individual level, when we examined correlations between emotion recognition performance and an eyes-mouth difference score, consistent with prediction #3 we found that for OA, more mouth exploration (relative to eye exploration) was associated with faster recognition of happy and disgusted faces. No associations were found between eye-mouth time and accuracy in either OA or YA. That these correlations were only found in the OA group, and not the YA group, is noteworthy, especially in the context of some previous eye tracking studies that showed relationships between viewing patterns and recognition performance in both YA and OA, at least for some emotions [12,13]. One possibility is that aspects of the experimental design did not permit variable enough performance in the YA group in order for these associations to be revealed at the individual level. For instance, in the case of Disgusted faces, the YA group showed somewhat restricted variance in eyes-mouth scores, whereas there was much more variability in eye-mouth scores in the OA group (See Fig 5) reflecting the OA group’s relative tendency to explore the mouth. For Happy faces, variance in RT in the YA group was limited, as young adults recognized happy faces very rapidly. Thus, it is possible that had the task been more difficult, increasing the variance in RTs and possibly also exploration patterns used to uncover expression information, correlations between eyes-mouth and performance would have been observed in younger adults.

When these somewhat disparate results are taken together, they suggest that in addition to attentional biases that may impact emotion recognition in OA to a certain degree, there is likely a genuine perceptual deficit that impedes this aspect of face perception from both eye and mouth information. That is, it may be that for at least some OAs, exploring the diagnostic features (e.g., the mouth in disgusted expressions) does not facilitate recognition of that emotion. This possibility is partially supported by Sullivan et al. [12], who found a negative (although not significant) association between mouth viewing time and overall accuracy, indicating that although OA viewed the mouth more, they were not able to use the information adequately to facilitate expression recognition. What is driving this age related perceptual deficit? The notion that a general age-related cognitive decline is responsible for difficulties with emotion recognition is not supported by the literature [2]. In addition, a general cognitive decline account would not account for the finding, here and elsewhere [2], that emotion recognition in OA is spared for some emotions but not for others. One possibility is that age related changes in frontal and temporal brain regions (e.g., amygdala) are responsible for reduced emotion recognition ability of specific emotions such as anger, fear and sadness [2, 31, 32, 33]. Interestingly, the basal ganglia, a subcortical area involved in disgust recognition, appears to be less affected by age [34, 35], which could explain why previous studies have found preserved disgust recognition, but does not explain why the present study found the largest age difference in recognition of disgust. In the context of the MWT, it could be that frontal networks involved in working memory are important to successful emotion recognition, because observers were forced to inspect one facial feature at a time, building a representation of the face over the course of the trial, instead of being allowed to process the face holistically (see Limitations). However, it is not clear why age-related changes in working memory would impact disgust recognition in the MWT selectively more than recognition of other expressions, and this explanation also does not fit with the evidence that emotion recognition impairments due to aging are unaccounted for by general cognitive decline [2]. Future research efforts should continue to clarify the neural mechanisms leading to specific patterns of emotion recognition impairment in aging adults [2].

We were secondarily interested in whether or not an age-related reduction in eye exploration might be limited to one eye over another. When we examined percentage of exploration time, we found that OA showed a selective reduction in exploration of the left eye (i.e., the eye in the LVF of the participant). This extends the work of a previous study using the MWT [24], in which a developmental increase was observed in left-eye exploration from childhood (aged 5–12 years) to young adulthood, resulting in a significant left-eye bias (greater exploration of the left eye than the right eye) in YA but not in children. We interpreted these results as suggesting that sometime after the age of 11–12 years, a shift occurs in attentional allocation to the left eye that may be related in part to maturation of right-lateralized face processing areas in the brain [27–30]. While not a central goal of the study, it is notable that the left eye bias was significant in both YA and OA, despite the OA group spending less time exploring the left eye relative to YA. Thus, the mechanisms driving the left eye bias in young adulthood appear to be intact in OA, albeit slightly weaker.

Despite finding that overall exploration time differences in OA were largely restricted to the left eye, when we examined the last ROI to be explored (i.e., at the time of response), we found that OA were less likely than YA to end their exploration on the right eye, and were more likely than YA to end their exploration on the nose. This result may reflect a decreased reliance on the upper parts of the face, and increased reliance on the lower parts of the face, when making an emotion judgment. However, inconsistent with this interpretation is that OA and YA were equally likely to end their exploration on the mouth. Thus, once again we find only partial support for our first prediction that, relative to YA, OA would spend less time on the upper parts of the face and more time on the lower parts of the face. In addition, we must exercise caution in interpreting the last ROI to be explored as an important area for emotion judgment, since as noted in the introduction, emotion recognition may occur rapidly, before the end of the trial.

Notably, both groups explored the mouth proportionally longer than any other part of the face, consistent with previous work using the MWT [24], but inconsistent with the broader eye tracking literature on attention allocation to faces. That is, much of the previous eye tracking research has shown a general bias to attend to the eyes over the mouth [6, 12, 36], particularly for expressions in which diagnostic information resides in the upper part of the face (e.g., [4]). In the NimStim faces that we presented to participants, mouths were open and highly expressive, which may have made them unusually informative for emotion recognition, even for expressions that are traditionally considered “top expressions”. For instance, while we found the expected pattern of more mouth exploration on happy faces than on angry and fearful faces, we found no difference in mouth exploration on disgusted and fearful faces, which would have been predicted based on previous work (e.g., [4, 7, 8, 24]). The overall strong bias to explore the mouth suggests that our stimulus set may provide an example of faces in which the mouths provide essential information for distinguishing between different expressions, blurring the distinction between “top” and “bottom” emotions. Alternatively, it may be that the MWT leads to different attentional biases than eye tracking techniques, because observers are forced to explore the face using a small window. Likewise, that the nose was also a highly explored region (second only to mouths) could suggest that this facial feature is also more informative for emotion recognition than previously thought, or that the nose is an important landmark that is used to guide the face exploration when using the MWT. Indeed, our analysis of the last ROI to be explored revealed that OA in particular may find the nose to be particularly helpful for emotion recognition (see also [16], who found increased fixation on noses in OA during an identity recognition task]. It will be important for future studies to make more direct comparisons between eye tracking and the MWT for revealing how attention is allocated to faces during emotion recognition across paradigms.

### Limitations

A limitation of the MWT is that it forced both groups to conduct an analytic exploration viewing strategy, in which individual features are inspected one at a time, eliminating the opportunity for holistic face processing (i.e., integrating information from the entire face simultaneously). In a recent eye tracking study, Chan, Chan, Lee & Hsiao (in press [37]) found that OA were more likely than YA to use a holistic viewing strategy during face recognition and that this approach correlated with worse face recognition across both groups, and also with cognitive decline in the older adults. Specifically, older adults’ fixations landed mainly around the face center, whereas YA utilized a more analytic strategy in which eye movements were distributed among the two eyes and face center [37]. Thus, it is possible that by using the MWT in our study, group differences in exploration strategy were artificially reduced, potentially weakening the conclusions that can be drawn. Alternatively, to the extent that recognition of facial expressions relies on both analytical and holistic processing of faces [38], if OA rely more on holistic information for emotion recognition, the MWT may have put them at a disadvantage. Although Chan et al.’s [37]) study focused on identity recognition, rather than emotion recognition, the reported age related difference in exploration patterns is particularly important when situating our findings relative to previous eye tracking studies that did not require participants to explore features of the face with a small window. However, while the MWT may have artificially forced both groups to employ an analytic viewing strategy, an aforementioned advantage of the MWT is that it allowed us to compare *which* ROIs are analytically inspected by OA vs. YA during emotion recognition when using such a strategy. Here the findings were clear: OA were less likely than YA to analytically explore the eyes, and marginally more likely to explore the nose, and mouth. These patterns as a whole are largely consistent with the previous eye tracking literature that did not force observers to inspect facial features through a small window, e.g., [10, 11, 12, 13]).

In addition, as mentioned previously, some of the face stimuli were not optimal in conveying the traditional distinction between “top” and “bottom expressions”, especially since the mouth was so highly expressive across all emotions. In addition, it is possible that because we included fairly intense expressions, emotion recognition was less dependent on attentional patterns, as opposed to more subtle expressions (e.g., [7]). This could have led to findings that do not reflect how attention supports emotion recognition for more subtle, everyday expressions of emotion. A replication of the study with the use of less expressive faces and those with closed mouths would shed light on this possibility. It is also important to consider other features of the study that could have potentially biased our results. For instance, it is well known that we are better at recognizing the identity of faces within our own age group [39] relative to other-age faces. This own-age effect for face recognition is thought to reflect more extensive recent experience with members of one’s own age group [40], or increased motivation to attend to faces of one’s own age group [41]. Since the face stimuli we used were all of young adults, it is possible that older adults were at a disadvantage, which could have affected our results. However, evidence that the own-age bias extends to decoding facial expressions is weak, with findings instead indicating that both younger and older participants are better at recognizing emotions from younger faces (e.g., [41, 42, 43]). In addition, Ebner & Johnson (2011) found that while observers spent more time looking at own-age than other-age faces, scanning patterns within the face did not show any evidence of an own-age effect [42]. Another consideration is that we did not pre-screen older adults for their proficiency at using a computer mouse, which one might reasonably expect to be affected by age or physical ailments (e.g., arthritis) associated with age, and to slow down search times. Furthermore, individual differences in response strategy (e.g., using one hand to manipulate the mouse and another to use the keyboard, vs. using the same hand for all responding) were not monitored and could also be impacted by age, contributing to more variable response times and exploration strategies in the OA group. While we acknowledge this as a potential limitation of our study, the heat maps and overall exploration patterns revealed relatively subtle differences in exploration patterns between OA and YA, with both groups spending most of their time exploring the internal features of the face, and differences in exploration time were restricted to these internal features (eyes, nose and mouth). Thus, we do not think that the patterns we observed can be accounted for by an age-related impairment in ability to explore the face with the handheld mouse. If anything, emotion recognition accuracy was quite high for both groups—above 85%. Indeed, the high levels of accuracy could have masked some of the expected group-level differences in performance, as well as restricting our ability to reveal associations between accuracy and eye vs. mouth exploration.

Finally, it is important to acknowledge that ROI-based approaches have been met with criticism for introducing experimenter bias, both with respect to the selection of ROIs to analyze, as well as the generation of these ROIs (e.g., should the eyes be analyzed as one unit? Or two, as we did in the present study? Should the eyebrows be included or not?). There has been a recent trend towards more data-driven approaches, such as eye movement analysis with hidden Markov models (EMHMM; [44]), which models person-specific ROIs and transitions among these ROIs, and then clusters individual HMMs to discover common patterns across individuals. This approach has the advantage of capturing individual differences in exploration strategy that can be correlated with other measures of performance [37]. Another powerful data-driven approach is iMap [45], which generates ROIs from fixation density maps, and computes group differences in viewing behaviour based on statistically robust fixation map subtractions. While we presented exploration heat maps for visualization purposes (see Fig 3), these maps were used to complement our ROI-based approach, rather than replace it. Despite the utility of these data-driven approaches, it is worth pointing out that we selected ROIs *a priori* because we had very specific predictions about how aging would affect exploration of these regions based on the aging eye tracking literature that used ROI-based analyses (e.g., [10, 11, 12, 13]) and previous studies that used data-driven techniques (e.g., “Bubbles”, [8]) to identify these specific ROIs as diagnostic for facial emotion recognition. Future studies could employ data-driven or hypothesis-free approaches to extend our findings.

### Conclusions

The present study uses the MWT to extend previous findings of aging effects on viewing behavior. Our findings suggest that attentional differences on their own are unlikely to explain age-related declines in emotion recognition performance, and that perceptual deficits are also at play. Although exploring faces using MWT may not accurately represent how faces are processed in the real world, MWT offers some advantages over eye tracking and gives a more direct measure of how attention is allocated in expression recognition.

## Supporting information

S1 Table SPSS spreadsheet containing inter-participant means for Accuracy (proportion correct), RT (sec), and percentage exploration time broken down by emotion condition (angry, fearful, happy, disgusted) and ROI (eyeL, eyeR, nose, mouth, face remainder, hairline/ears). Subject IDs are numeric, Age (1 = YA, 2 = OA).
(CSV)

S2 Table SPSS spreadsheet containing inter-participant means for percentage of trials on which exploration ended (i.e., position of the moving window at the end of the trial) on the left eye, right eye, nose, or mouth, broken down by emotion condition (angry, fearful, happy, disgusted). Subject IDs are numeric, Age (1 = YA, 2 = OA).
(CSV)
