# Correction: Exploring emotional expression recognition in aging adults using the Moving Window Technique

**DOI:** 10.1371/journal.pone.0208767

**Published:** 2018-12-04

**Authors:** The PLOS ONE Staff

## Notice of republication

Fig 1 was published in error. This article was republished on 21 November 2018, to correct Fig 1. The publisher apologizes for the error. The correct figure is available in the Supporting Information of this article.

## Supporting information

S1 File Corrected Fig 1. (TIF)
