# Supplementary figures and images for: Correction: Exploring emotional expression recognition in aging adults using the Moving Window Technique
Source: PLoS One. 2018 Dec 4;13(12):e0208767. doi: 10.1371/journal.pone.0208767 (PMC6279025; doi:10.1371/journal.pone.0208767)

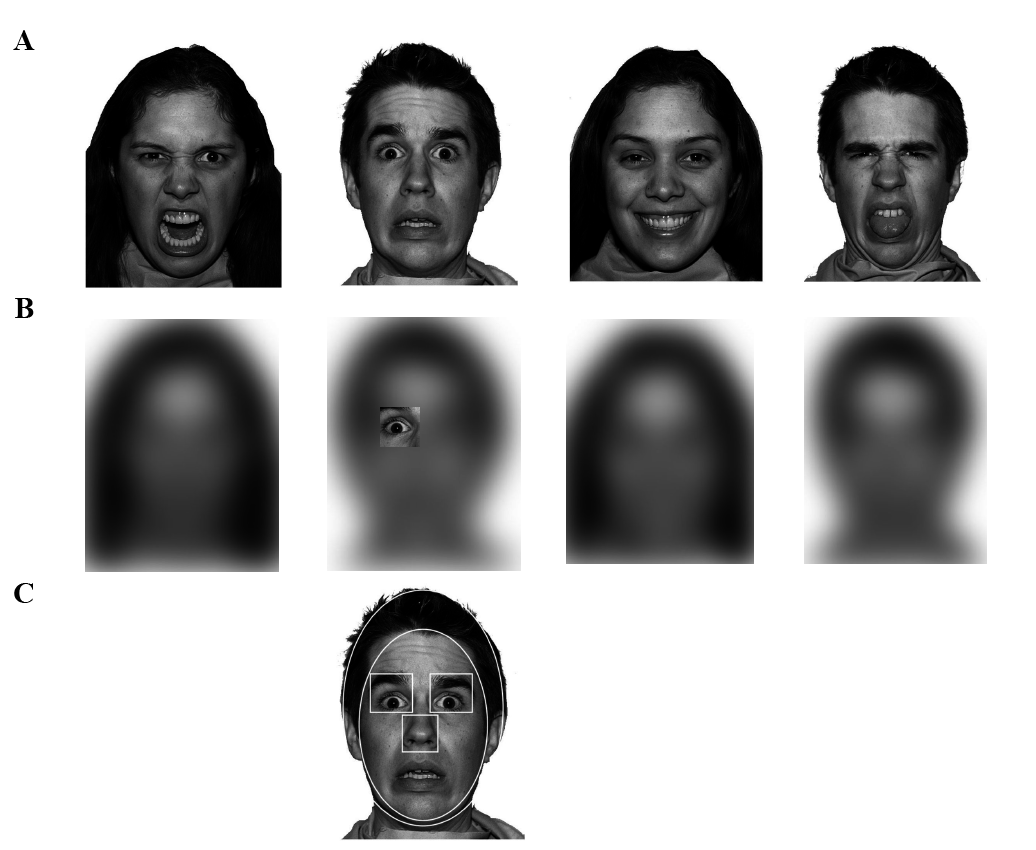

Supplement: S1 File — (TIF) [file pone.0208767.s001.tif]
